# Cell-type-specific cis-eQTLs in pancreatic cell types identify novel risk genes for type 2 diabetes

**DOI:** 10.1093/bib/bbaf531

**Published:** 2025-10-09

**Authors:** Xiao-Cao Miao, Hui Li, Qing Li, Lei Zhu, Yan-Qiu Yu, Jian-Guang Ji, Tao Chen, Zhi-Gang Zhang, Dong-Xue Li

**Affiliations:** State Key Laboratory of Systems Medicine for Cancer, Shanghai Cancer Institute, Ren Ji Hospital, School of Medicine, Shanghai Jiao Tong University, 800 Dongchuan Road, Minhang District, Shanghai 200240, PR China; State Key Laboratory of Systems Medicine for Cancer, Shanghai Cancer Institute, Ren Ji Hospital, School of Medicine, Shanghai Jiao Tong University, 800 Dongchuan Road, Minhang District, Shanghai 200240, PR China; State Key Laboratory of Systems Medicine for Cancer, Shanghai Cancer Institute, Ren Ji Hospital, School of Medicine, Shanghai Jiao Tong University, 800 Dongchuan Road, Minhang District, Shanghai 200240, PR China; State Key Laboratory of Systems Medicine for Cancer, Shanghai Cancer Institute, Ren Ji Hospital, School of Medicine, Shanghai Jiao Tong University, 800 Dongchuan Road, Minhang District, Shanghai 200240, PR China; Department of Pathophysiology, School of Basic Medicine, China Medical University, No. 77 Puhe Road, Shenbei New District, Shenyang 110122, Liaoning Province, PR China; Shenyang Engineering Technology R&D Center of Cell Therapy Co., Ltd, No. 400-8, 2nd Zhihui Street, Hunnan District, Shenyang 110174, Liaoning Province, PR China; Department of Public Health and Medicinal Administration, Faculty of Health Sciences, University of Macau, S22 Avenida da Universidade, Taipa, Macau SAR 999078, PR China; Cancer Center, Faculty of Health Sciences, University of Macau, S22 Avenida da Universidade, Taipa, Macau SAR 999078, PR China; Department of Gastrointestinal and hernia surgery, Ganzhou Hospital-Nanfang Hospital, Southern Medical University, No. 16 Meiguan Avenue, Zhanggong District, Ganzhou, Jiangxi 341000, PR China; Department of General Surgery & Guangdong Provincial Key Laboratory of Precision Medicine for Gastrointestinal Tumor, Nanfang Hospital, Southern Medical University, No. 1838 North Guangzhou Avenue, Baiyun District, Guangzhou, Guangdong 510000, PR China; State Key Laboratory of Systems Medicine for Cancer, Shanghai Cancer Institute, Ren Ji Hospital, School of Medicine, Shanghai Jiao Tong University, 800 Dongchuan Road, Minhang District, Shanghai 200240, PR China; State Key Laboratory of Systems Medicine for Cancer, Shanghai Cancer Institute, Ren Ji Hospital, School of Medicine, Shanghai Jiao Tong University, 800 Dongchuan Road, Minhang District, Shanghai 200240, PR China

**Keywords:** type 2 diabetes, cell type specific, cis-eQTLs, eGenes, single-cell RNA-sequencing, chromatin accessibility

## Abstract

Type 2 diabetes (T2D) is a complex metabolic disorder strongly influenced by genetics. Most genetic studies, including expression quantitative trait loci (eQTL) analyses, use bulk pancreatic tissue, masking cell-specific mechanisms. Here, by integrating single-cell RNA sequencing, chromatin accessibility, and genome-wide association studies (GWAS) data, we systematically identified 328 pancreatic cell-type-specific cis-eQTLs associated with T2D. We pinpointed nine key genes (including *STIL* in beta and delta cells; *ZSWIM5* in alpha, delta, and ductal cells; *IL1RN*, *ANP32E*, *IPP*, *MLLT11*, and *SLC23A3* in delta cells; *SNX4* in gamma cells; and *RBMS1* in beta cells) whose SNPs overlapped with chromatin accessibility peaks. These genes highlight regulatory pathways in beta-cell dysfunction, metabolic stress responses, and disrupted pancreatic homeostasis. A public database, CTPeQTLs (https://ctpeqtls.netlify.app/), was developed to explore cis-eQTLs across diabetic and non-diabetic cohorts, revealing distinct regulatory patterns in both endocrine and exocrine cells, as well as disease-associated transcriptional dysregulation. Our findings uncover cell-specific genetic mechanisms in diabetes and provide potential therapeutic targets, supporting precision medicine strategies.

## Graphical Abstract

**Graphical Abstract:**
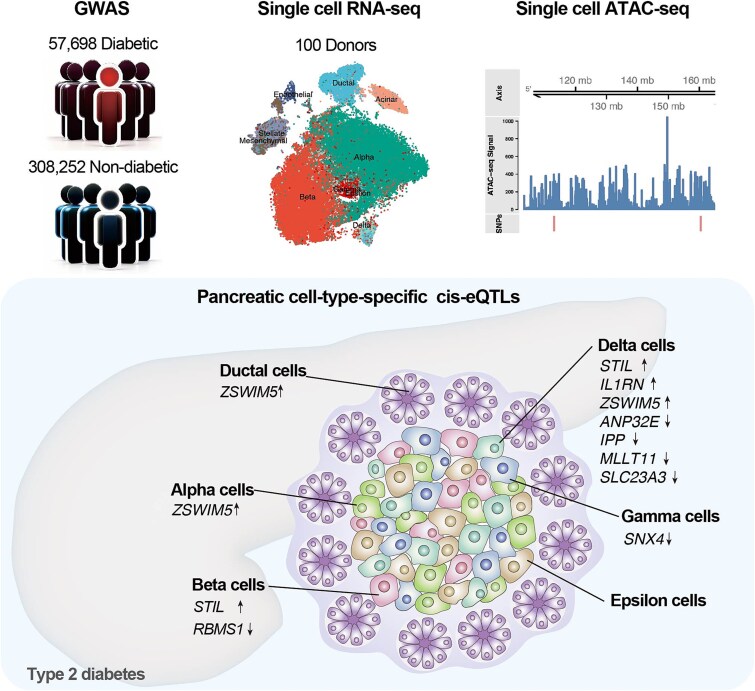

## Introduction

Type 2 diabetes (T2D) is recognized as one of the foremost global health challenges, characterized by chronic metabolic dysfunction affecting millions worldwide and imposing a heavy burden on healthcare systems [1, 2]. At its core, T2D involves the progressive deterioration of insulin secretion capacity from pancreatic β cells, often accompanied by insulin resistance [3]. Despite decades of extensive research, the precise molecular and genetic underpinnings of T2D remain incompletely defined.

Genetic factors undeniably contribute significantly to T2D susceptibility, with heritability estimates exceeding 50% [4–6]. GWAS have substantially advanced our understanding of diabetes genetics, successfully identifying over 700 genetic loci linked to increased T2D risk [6–10]. However, GWAS typically survey genetic variation across the entire genome. As a result, most identified loci consist of single nucleotide polymorphisms (SNPs) situated predominantly within non-coding genomic regions. These regulatory regions may influence cellular states and disease progression through epigenetic mechanisms, such as chromatin accessibility and DNA methylation [11]. Moreover, each individual SNP often exhibits only a modest effect size, posing a substantial challenge when attempting to pinpoint their exact biological roles [12]. Consequently, elucidating how these non-coding variants exert their effects, and identifying the specific genes they regulate, remain pressing priorities in diabetes research.

Expression quantitative trait loci (eQTL) analysis, a method linking genetic variants directly to gene expression levels, provides a valuable pathway to decipher the functional relevance of genetic variants [13]. Although eQTL approaches have been extensively employed in diverse tissues and disease contexts, T2D-focused eQTL studies to date have mainly analyzed bulk pancreatic tissue, largely neglecting critical differences across distinct cell types [14–18]. Recent advances in single-cell RNA sequencing (scRNA-seq) offer unparalleled opportunities to address this limitation by dissecting genetic regulation at the level of individual cells, particularly given the considerable functional heterogeneity within pancreatic cell populations, such as alpha, beta, and ductal cells [19–22].

In this study, we address these knowledge gaps by integrating GWAS data, single-cell RNA sequencing, and assay for transposase-accessible chromatin with high throughput sequencing (ATAC-seq). For the first time, we systematically perform cis-eQTL analysis at single-cell resolution in human pancreatic cells. By narrowing the broad spectrum of GWAS-identified SNPs to those specifically influencing gene expression within distinct pancreatic cell types, our analysis greatly improves the precision of genetic insights into T2D pathogenesis. Notably, we identify 328 pancreatic cell-type-specific eGenes associated with T2D, uncovering substantial cell-type specificity and distinct gene regulatory patterns between diabetic and non-diabetic individuals. Furthermore, we highlight genetic variants that directly overlap with chromatin accessibility regions, providing mechanistic clues into how non-coding SNPs potentially modulate gene expression and ultimately influence T2D risk.

Taken together, our findings substantially advance our understanding of T2D genetics by illuminating previously unrecognized cell-type-specific genetic regulatory mechanisms in the pancreas. These insights offer critical foundations not only for unraveling the complex pathogenesis of T2D but also for informing the development of precise therapeutic interventions tailored toward individual genetic profiles.

## Material and methods

### GWAS summary statistics

We obtained a total of 92 distinct publicly available GWAS summary statistics for diabetes from the IEU Open GWAS Project database [23], FinnGen database [24], and the GWAS Catalog database [25]. In the case group, diabetes of unknown types was excluded, and only T2D cases were retained. The numbers of T2D cases in each group were compared, and the finngen_R10_T2D from the FinnGen consortium R10 release dataset, with the largest number of cases (57 698 diabetic and 308 252 non-diabetic individuals), was selected. The summary statistics were used to perform downstream analyses, including summary-data-based Mendelian randomization (SMR) and colocalization analyses.

### eQTL datasets

We acquired four peripheral blood eQTL summary datasets from major consortia: the eQTLGen Consortium (*N* = 31 684) [26], Westra *et al*. (*N* = 3511) [27], CAGE (*N* = 2765) [28], and GTEx v8 (*N* = 670) [29]. Based on sample size considerations, we selected the eQTLGen Consortium dataset for subsequent analyses as it provides the largest statistical power for eQTL detection.

### Human eQTL analysis

The test for pleiotropic association between the expression level of a gene and a complex trait of interest was conducted using summary level data from GWAS and eQTL studies by the SMR software tool [30]. Detailed information regarding the SMR method has been described previously. Heterogeneity testing was conducted using up to the top 20 SNPs within the cis-eQTL region, including the top cis-eQTL. No filtering of SNPs was performed based on the minor allele frequency. The threshold for the difference in allele frequencies was set at 0.2, and the maximum proportion of SNPs with allele frequency differences that was permitted was 0.05. The p-value threshold for selecting the top associated eQTLs for the SMR test was 5.0e-8. The p-value threshold for selecting eQTLs for the Heterogeneity in dependent instruments (HEIDI) test was 1.57e-3, which corresponded to a chi-squared value of 10. The window size for defining the selection of cis-eQTLs around the probe was set at 2000 kilobases (Kb). The minimum number of cis-SNPs used in the HEIDI test was 3, and the maximum number of eQTLs used was 20. A final set of 371 unique eGenes was derived by mapping significant SNPs (FDR < 0.05) to their target genes and removing duplicate gene-level entries.

### Single cell RNA-seq datasets

We integrated scRNA-seq data from ten published studies in the Gene Expression Omnibus (GEO) and ArrayExpress databases. The E-MTAB-5061 dataset includes 4 diabetic and 6 non-diabetic individuals [31]. The GSE101207 dataset includes 3 diabetic and 6 non-diabetic individuals [32]. The GSE124742 dataset includes 7 diabetic and 3 non-diabetic individuals [33]. The GSE153855 dataset includes 5 diabetic and 6 non-diabetic individuals [34]. The GSE154126 dataset includes 10 diabetic and 4 non-diabetic individuals [35]. The GSE195986 dataset includes 4 diabetic and 7 non-diabetic individuals [36]. The GSE81608 dataset includes 6 diabetic and 12 non-diabetic individuals [37]. The GSE83139 dataset includes 2 diabetic and 3 non-diabetic individuals [38]. The GSE84133 dataset includes 1 diabetic and 3 non-diabetic individuals [39]. The GSE86473 dataset includes 3 diabetic and 5 non-diabetic individuals [40].

### Quality control and the dimensionality reduction of single-cell RNA-seq datasets

The gene expression data were loaded into the Seurat (version 5.0.1) [41] R (version 4.2.1) package for analysis. The gene-cell matrices underwent filtering to remove cells with fewer than 500 transcripts or more than 5% mitochondrial genes. For each sample, gene expression values were normalized by calculating the fraction of each gene’s expression relative to the total, scaled by a factor of 10 000, and then transformed using the natural logarithm after adding one to prevent taking the logarithm of zero. From the normalized expression matrix, the top 1000 highly variable genes were identified and used as input for principal component analysis (PCA). Dimension reduction was performed using the FindNeighbors and FindClusters functions, and the resulting clusters were visualized using t-distributed stochastic neighbor embedding (tSNE).

### Cell-clustering and annotation

To determine cluster-specific marker genes, the FindAllMarkers function in the Seurat package was employed, utilizing the default non-parametric Wilcoxon rank sum test with Bonferroni correction for statistical significance. The cell groups were annotated based on the differentially expressed genes (DEGs) and the well-known cellular markers from the literature (*INS* for beta cells, *GCG* for alpha cells, *SST* for delta cells, *PPY* for gamma cells, *GHRL* for epsilon cells, *COL1A1* for stellates, *PRSS1*/*CPA1* for acinar, *KRT19* for ductal cells, and *PECAM1* for endothelial cells).

### Single-cell data integration and batch effect correction

We performed comprehensive integration of 10 single-cell RNA-seq datasets (GSE195986, GSE83139, GSE154126, GSE153855, GSE124742, GSE101207, GSE84133, GSE81608, EMTAB5061, and GSE86473) using the Seurat integration pipeline. Each dataset was first processed independently through quality control, normalization using LogNormalize method, and cell type annotation. We identified 2000 highly variable features per dataset using the variance stabilizing transformation (VST) selection method, which were then used to determine integration anchors through the FindIntegrationAnchors function (dims = 1:30, anchor.features = 2000). The anchors were subsequently passed to the IntegrateData function to generate a batch-corrected integrated expression matrix containing all cells. Integration quality was quantitatively assessed using batch average silhouette width (ASW), which was computed with the silhouette function from the cluster package (version 2.1.8) [42]. Finally, the integrated data were clustered using default settings for downstream analysis.

### Cell-type-specific eQTL mapping

To identify cell-type-specific eQTLs, we integrated the eGene set with scRNA-seq data and constructed an eGene-restricted Seurat object. Cell types were assigned as described above. Rather than re-calling eQTLs in single-cell data, we quantified the cell-type enrichment of these GWAS/SMR-derived eGenes by computing per-cell eGene scores using single-sample gene set enrichment analysis (ssGSEA). ssGSEA was implemented with the GSVA R package (version 1.48.0; method = ‘ssgsea’) [43], using the union of T2D-associated eGenes as a single gene set; per-locus (per-eQTL) gene sets were not used because most loci map to one eGene and are ill-suited for ssGSEA. The resulting scores reflect the extent of genetic regulation of expression at the single-cell level. eGene score categories were defined by quartiles computed across all cells pooled from all datasets and conditions: low (≤Q1; 25th percentile), medium (Q1–Q2; 25th–50th percentile), high (Q2–Q3; 50th–75th percentile), and very high (>Q3; >75th percentile). Scores were clustered by cell type to assess specificity.

To avoid pseudo-replication, the group comparisons of the number of cis-eQTL per single cell and the eGene score were conducted at the donor level: for each donor × cell type, per-cell ssGSEA scores were summarized by the mean, and donor–cell-type strata with fewer than 20 cells were excluded. Diabetic and non-diabetic groups were compared using two-sided Wilcoxon rank-sum tests on the donor-level summaries; *P*-values were Benjamini–Hochberg adjusted where multiple cell types were tested. Boxplots display the median (center line), interquartile range (box), and 1.5 × IQR whiskers, and the number of donors (n) per group is annotated on each panel. Results were visualized with ggplot2 (version 3.4.2) and Seurat (version 5.0.1) [44].

### Cell-type-specific functional enrichment analysis

To identify molecular functions associated with highly and lowly expressed eGenes in each distinct cell type, we performed Gene Ontology (GO) enrichment analysis. The analysis was conducted using the clusterProfiler package (version 4.8.1) in R [45]. Gene sets were defined according to the expression levels of eGenes. Within each cell type, genes with an average log_2_fold change (avg log2FC) > 0.25 and an adjusted *P*-value (*P* val adj) < .05 were defined as highly expressed genes, while genes with an avg log2FC < −0.25 and a *P* val adj < .05 were defined as lowly expressed genes. Differential expression analysis was performed using ​​limma​​ (version 3.54.0) [46]. The results were visualized using the ggplot2 package (version 3.4.2). To address potential biases from differential cell counts, we performed correlation analysis between cell ratio differences (|1 – diabetic/non-diabetic cell ratio| × 100) and the number of DEGs for each cell type. Statistical significance was assessed using Pearson correlation with α = 0.05. The GO enrichment analysis was performed using the enrichGO function, with the following parameters: pvalueCutoff was 0.05 and qvalueCutoff was 0.05. To account for multiple testing, p-values were adjusted using the Benjamini–Hochberg (BH) method, and GO terms with an FDR < 0.05 were considered statistically significant. The results were visualized using the dotplot and enrichMap functions from the clusterProfiler package to highlight the most enriched GO terms and their relationships. Enriched terms were grouped by ontology (GO BP/MF/CC) and KEGG, and we displayed the top five terms per category ranked by BH-adjusted *P* values (FDR) to improve readability. Dot color encodes −log10 (FDR), and dot size encodes the gene ratio (k/n, where k is the number of eGenes in the term and n is the total number of eGenes tested for that panel). Selected pancreas-relevant pathways (e.g. insulin secretion/signaling, lipid metabolism, ER/oxidative stress, vesicle trafficking) were visually emphasized. Complete statistics (FDR, gene ratio, z score, background size, leading-edge genes) are provided.

### Data processing of single cell ATAC-seq signals and SNP overlaps

We used single-cell ATAC-seq data from GSE194401 to assess chromatin accessibility in pancreatic cell types, which includes 4 diabetic and 7 non-diabetic individuals [47]. The dataset included BigWig files representing chromatin accessibility signals for distinct cell types. BigWig files for each cell type were imported into R using the import function of the rtracklayer (version ​1.62.0) package [48]. SNP information was obtained from the dbSNP database, specifically the dbsnp_146.hg38.vcf.gz file for the hg38 genome assembly. The VCF file was read using the readVcf function of the VariantAnnotation (version 1.48.0) package, with the genome parameter set to ‘hg38’ [49]. The set of cell-type-specific eQTLs was defined for each cell type, the genomic coordinates of these SNPs were extracted from the VCF file using the rowRanges function and filtered to include only the SNPs of interest. The genomic coordinates of these SNPs were extracted from the VCF file using the rowRanges function of the GenomicRanges (version 1.54.0​) package and filtered to include only the SNPs of interest [50]. Visualization was performed using the Gviz (version 1.46.0) package to create tracks for the ATAC-seq signals and SNP annotations [51].

### Colocalization analysis

We performed colocalization analysis to assess the relationship between eQTL effect sizes and GWAS effect sizes for SNPs associated with the identified risk genes. We tested colocalization between the GWAS and eQTL signals for SNPs with corresponding identified risk genes using the ‘coloc.abf’ function of the coloc R package (version 5.1.0) [52]. SNPs with a posterior probability of colocalization (PPC) > 0.75 were considered significant. The results were visualized using the ggplot2 (version 3.4.2) package.

### Expression levels of eGenes in pancreatic disease models​

Gene expression profiles for the nine prioritized eGenes were systematically extracted from the Cancer Cell Line Encyclopedia (CCLE) database [53]. The analysis encompassed four clinically stratified pancreatic disease categories: non-cancerous pancreas (ABMT9430), pancreatic neuroendocrine tumor (QGP1), adenosquamous carcinoma (KP3, L33), and 51 pancreatic adenocarcinoma lines (PATU8988T, PATU8988S, PANC1, PANC0213, T3M4, etc.). All cell lines were maintained under rigorously standardized conditions.

### CTPeQTLs database implementation​​

To facilitate interactive exploration of pancreatic cell-type-specific cis-eQTLs in both diabetic and non-diabetic cohorts, we developed the publicly accessible CTPeQTLs database (https://ctpeqtls.netlify.app/). The database is built on a lightweight yet scalable architecture, featuring a vanilla HTML/CSS/JavaScript front end integrated with Plotly.js for real-time and interactive data visualization. The back end stores gene expression values, cell metadata, and t-SNE coordinates in an optimized sparse matrix format to ensure rapid query performance. Users can dynamically retrieve and visualize results via dual dropdown selectors, filter by cell type and disease status, and toggle between raw and aggregated data views. The platform is designed to be periodically updated as new datasets become available, with a version log maintained to document all content changes. This architecture ensures long-term usability, facilitates reproducibility, and provides a foundation for integration with future multi-omics analyses.

## Results

### Identification of type 2 diabetes associated cis-eQTLs

To pinpoint genetic variants responsible for regulating gene expression in specific pancreatic cell types associated with T2D, we collated 92 distinct GWAS datasets related to diabetes from the IEU Open GWAS Project database [23], the FinnGen database [24], and the GWAS Catalog database [25] (Table S1). Among these, we selected the finngen_R10_T2D dataset, which contained the highest number of T2D cases, encompassing 57 698 T2D cases and 308 252 controls. Subsequently, we conducted SMR analyses of eQTL dataset from eQTLGen Consortium with 31,684 individuals [26].

Our SMR/HEIDI (heterogeneity in dependent instruments) analyses identified 364 SNPs demonstrating consistent causal associations across the eQTLGen Consortium cis-eQTL datasets (Fig. S1). These SNPs represented approximately 0.004% of the total SNPs analyzed within the dataset and corresponded to 371 eGenes (Table S2). Interestingly, these eGenes were predominantly located on chromosomes 1, 2, and 3 (Fig. S2).

### Type 2 diabetes and non-diabetic pancreatic cell profile

**Figure 1:**
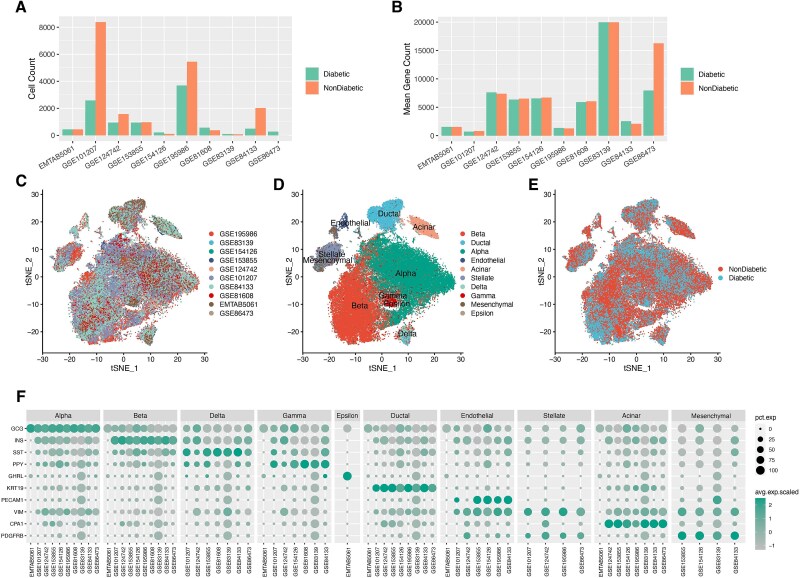
Distribution and characterization of pancreatic cells from 55 non-diabetic and 45 T2D organ donors, including cell counts across ten scRNA-seq datasets (A), mean gene counts (B), and t-SNE visualizations colored by dataset (C), cell type (D), and health status (E), as well as marker gene expression (F).

We obtained read count tables for ten single-cell human pancreatic datasets [E-MTAB-5061 [31], GSE101207 [32], GSE124742 [33], GSE153855 [34], GSE154126 [35], GSE195986 [36], GSE81608 [37], GSE83139 [38], GSE84133 [39], GSE86473 [40] (Table S3)], all of which included diabetic and non-diabetic donors. Metadata were downloaded from GEO, ArrayExpress, or publication supplements. After excluding cells with low gene expression and over 5% mitochondrial gene expression, the datasets were clustered with default settings, and then labeling was performed to identify different cell types of each of the ten datasets (Fig. S3). The datasets GSE101207 and GSE195986 contain more cells (Fig. 1A, Table S4) but detect fewer mean gene counts (Fig. 1B), consistent with their Drop-seq methodology as opposed to SMART-seq.

We merged the ten human pancreatic scRNA-seq datasets while preserving the originally annotated cell information. Following normalization, batch correction, and clustering, we generated a merged expression matrix, encompassing a total of 100 pancreatic tissue samples (45 from diabetic and 55 from non-diabetic individuals). To evaluate integration quality across heterogeneous platforms (notably Drop-seq versus SMART-seq), we calculated the ASW for batch labels. The batch ASW of −0.184 confirms robust integration, as values approaching zero indicate minimal batch effects. To visualize the distribution and relationships of cell types, we performed t-distributed stochastic neighbor embedding (tSNE) analysis, with plots colored by dataset accession (Fig. 1C), cell type (Fig. 1D), and health status (diabetic or non-diabetic; Fig. 1E). The results showed high integration fidelity, with clear separation of biological signatures and minimal batch effects. A total of 10 distinct clusters were identified among 68 050 pancreatic cells. Based on established cell-type-specific marker expression (Fig. 1F), these clusters were categorized into endocrine populations (alpha, beta, delta, gamma, epsilon cells) and non-endocrine lineages (acinar, endothelial, mesenchymal, and stellate cells).

### Identification of pancreatic cell type cis-eQTLs

**Figure 2:**
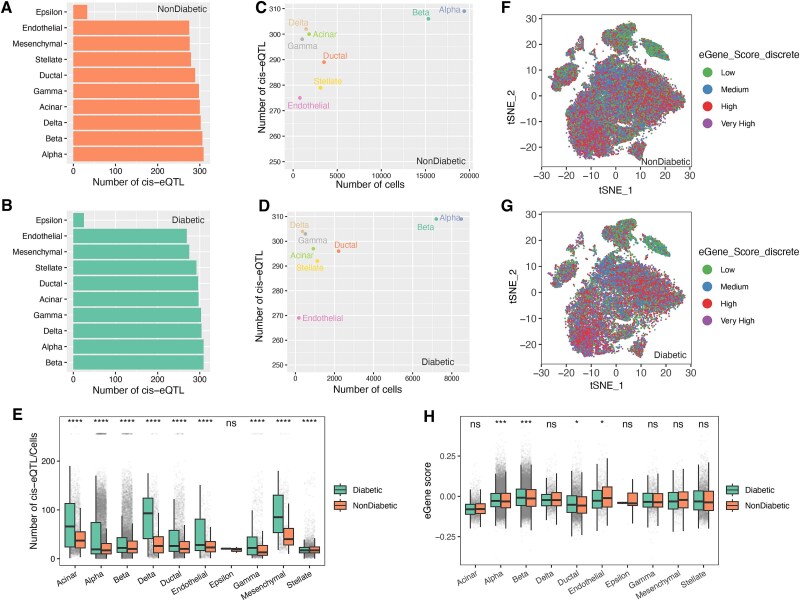
Cell-type-specific cis-eQTLs in pancreatic cells from diabetic and non-diabetic donors, including eQTL counts (A–B), relationships with single-cell numbers (C–D), comparisons of cis-eQTLs per cell (E), eGene score distributions by quartile (F–G), and group differences in eGene scores (H).

To identify cell type-specific cis-eQTLs in pancreatic islets, we integrated our curated eGene dataset with the aggregated single-cell transcriptomic profiles. This analysis revealed 328 robustly expressed eGenes across the integrated scRNA-seq dataset (Table S5), with 47 eGenes (14.3%) consistently shared across all 10 datasets (Fig. S4). We observed substantial variation in cis-eQTL detection rates across pancreatic cell types (Fig. 2A, B). Notably, beta cells exhibited 306 cis-eQTLs in non-diabetic samples compared to merely 33 in epsilon cells. Similarly, in diabetic samples, we detected 309 cis-eQTLs in beta cells versus only 25 in epsilon cells (Table S6). We observed high correlation between cis-eQTL discovery rates and the cellular abundance of each cell type (Fig. 2C, D), indicating that increased sampling depth enhances both gene expression quantification accuracy and statistical power for cis-eQTL detection. Furthermore, when comparing diabetic versus non-diabetic individuals, we found consistently elevated cis-eQTLs per cell in diabetic samples across nearly all cell types. This observation implies a potential broad-scale alteration or reduction in the regulatory efficiency of genetic variants influencing gene expression within diabetic pancreatic cells (Fig. 2E).

To further quantify cell-type-specific enrichment of eGenes, we applied ssGSEA to generate eGene scores based on gene expression profiles. Clustering analysis of these eGene scores revealed distinct regulatory patterns for each pancreatic cell type, emphasizing the specificity of genetic control over gene expression (Fig. 2F, G). Moreover, we observed that diabetic individuals consistently exhibited higher eGene scores compared to non-diabetic individuals, particularly in alpha, beta, and ductal cells, suggesting diabetes-associated alterations in the regulatory landscape controlling gene expression in these cells (Fig. 2H). In addition, we developed a publicly accessible platform, CTPeQTLs (https://ctpeqtls.netlify.app/), to facilitate browsing of eGenes in different pancreatic cell types.

### Pancreatic cell type eQTLs mediate type 2 diabetes associations

**Figure 3:**
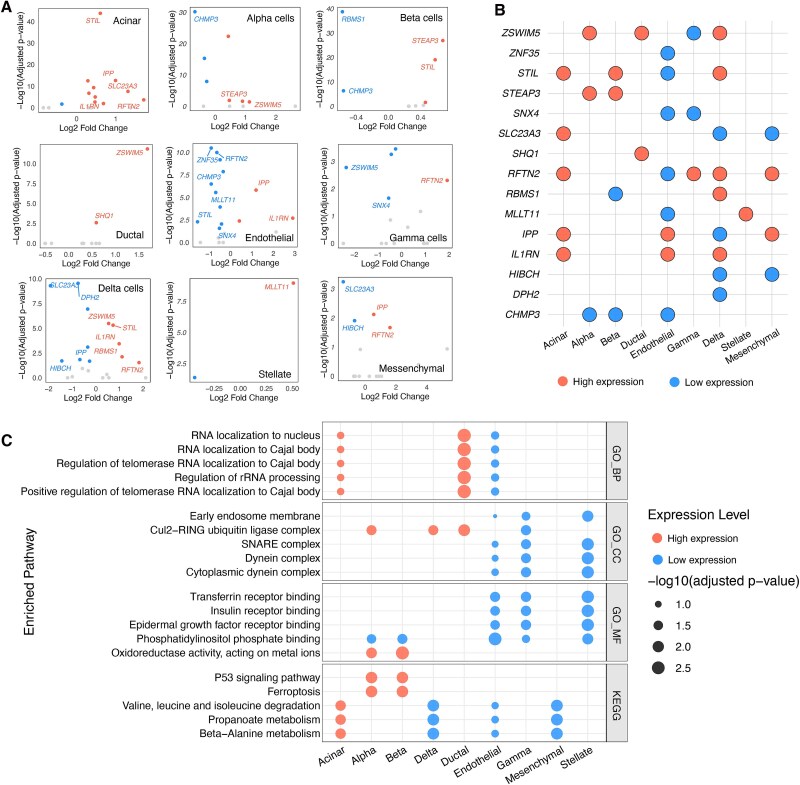
Differential gene expression and pathway enrichment in pancreatic cell types from diabetic versus non-diabetic donors, including cell-specific differentially expressed genes (A), summary of cell type–restricted dysregulation (B), and enriched GO/KEGG pathways (C).

To investigate pancreatic cell-type-specific gene expression in T2D, we analyzed scRNA-seq data from 55 non-diabetic and 45 T2D organ donors, identifying significantly DEGs in each cell type. Highly expressed DEGs in T2D versus non-diabetic were defined by average log2 fold-change (avg log2FC) > 0.25 and adjusted p-value (p val adj) < 0.05, while lowly expressed DEGs had avg log2FC ≤ 0.25 and *P* val adj < .05 (Figs 3A and S5). Distinct expression patterns clearly separated DEGs into highly or lowly expressed groups across cell types. Figure S6A shows post-QC counts per cell type split by diabetic and non-diabetic groups. Cell-ratio differences were not significantly correlated with the number of DEGs across cell types (Pearson r = 0.477, p = 0.19; Fig. S6B), suggesting that composition did not systematically inflate or attenuate DEG detection.

By aggregating cell-specific eGenes (Fig. 3B) and SNPs (Fig. S7) consistently highly or lowly expressed in diabetic individuals, we generated a summary plot enabling rapid visualization of dysregulated genes across pancreatic cell types in diabetes. Our analysis uncovered two principal categories of transcriptional dysregulation in diabetic pancreatic cells. First, broadly dysregulated genes showed consistent expression patterns across multiple cell types. For instance, *STIL* and *ZSWIM5* maintained elevated expression in acinar, alpha, and beta cells, likely participating in fundamental diabetes pathways including chronic metabolic stress responses and beta cell dysfunction. In contrast, *CHMP3* displayed widespread downregulation, potentially compromising critical regulatory functions in pancreatic homeostasis. Second, cell type specific signatures emerged, most notably the selective upregulation of *RBMS1* in beta cells. As an RNA binding protein, *RBMS1* may play specialized roles in preserving beta cell identity during diabetic progression. These stratified expression patterns collectively provide a multiscale perspective on diabetes pathogenesis, revealing both systemic pancreatic dysfunction and cell autonomous mechanisms. Chromosomal distribution analyses showed that both highly and lowly expressed genes were enriched on chromosomes 1, 2, and 3 (Fig. S8).

Through systematic GO analysis of biological processes (BP), cellular components (CC), and molecular functions (MF), along with Kyoto Encyclopedia of Genes and Genomes (KEGG) pathway analysis of cell type-specific DEGs (|avg_log2FC| > 0.5, adjusted p < 0.05), we identified distinct molecular signatures across pancreatic cell populations in T2D (Fig. 3C). ​​For enhanced clarity, Fig. 3C displays only the top 5 most significantly enriched pathways per functional category based on smallest adjusted p-value, with comprehensive statistical details including enrichment scores, gene ratios, and p-values documented in Table S7. Endocrine cells exhibited both shared and distinct pathological features. While alpha and beta cells both showed activation of ferroptosis and p53 pathways, indicating common stress responses, they displayed cell-type-specific functional impairments: transporter activity in alpha cells versus endocytosis in beta cells. Notably, the consistent downregulation of ESCRT III complex across endocrine cells suggests a pan-endocrine defect in vesicular trafficking that may contribute to diabetes progression.

Exocrine and stromal cells demonstrated both metabolic reprogramming and microenvironmental remodeling. The shared upregulation of RNA-related processes in acinar and ductal cells points to a common exocrine adaptation, while stromal cells uniformly exhibited stress responses, though through distinct mechanisms (immune activation in endothelial cells versus mitochondrial dysfunction in stellate cells). Importantly, the widespread dysregulation of metabolic pathways across all cell types highlights metabolic disturbance as a fundamental characteristic of the diabetic pancreas.

### Mapping of risk variants to cell type regulatory elements

**Figure 4:**
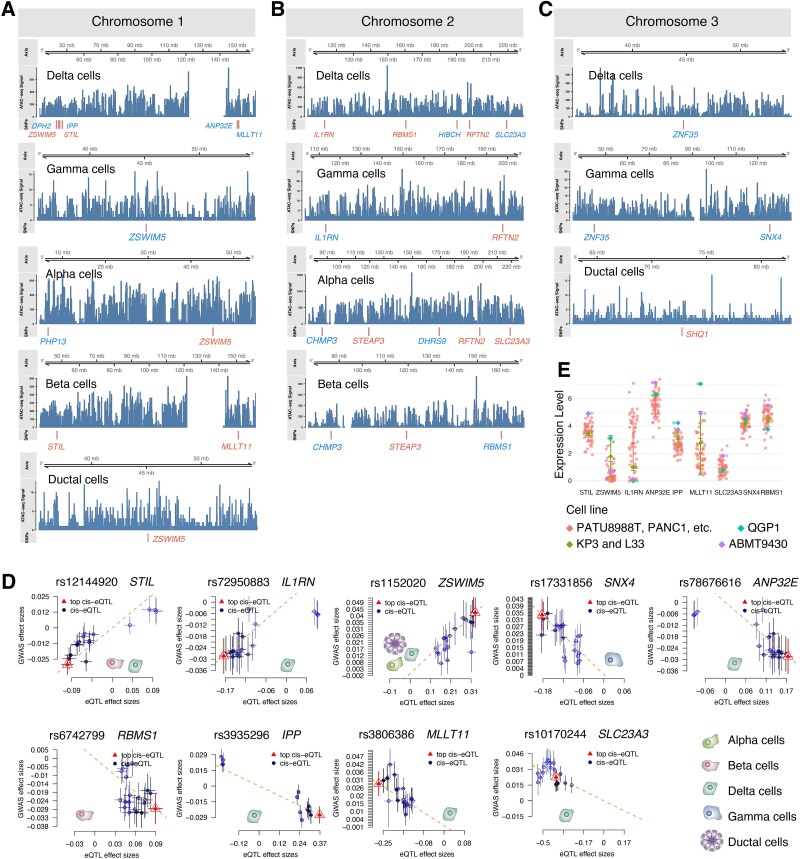
Epigenomic overlap of T2D GWAS SNPs with cell type–specific regulatory elements in pancreatic cells, including chromatin accessibility peaks in endocrine and ductal cells (A–C), relationships between GWAS and eQTL effect sizes (D), and expression levels of nine key eGenes in pancreatic disease-related cell lines (E).

To determine whether GWAS-identified SNPs overlap with regulatory regions near co-localized eGenes in specific pancreatic cell types, we integrated single-cell ATAC-seq data (GSE194401) [47] with both the significant eGenes identified in Fig. 3B and their associated SNPs from our analysis. Our chromatin accessibility analysis revealed that SNPs on chromosome 1 exhibited extensive overlap with accessible chromatin regions across all five pancreatic cell types (Fig. 4A), with particularly strong cell-type-specific overlaps observed for rs12144920 near *STIL* (beta and delta cells) and rs1152020 near *ZSWIM5* (alpha, delta, and ductal cells), suggesting these variants may regulate gene expression through modulation of transcription factor binding or chromatin remodeling. On chromosome 2, while delta and gamma cell SNPs generally co-localized with accessible chromatin, the *STEAP3* associated SNP rs3769653 showed no overlap with beta- or alpha-cell-specific peaks (Fig. 4B). Similarly, chromosome 3 analysis revealed distinct cell-type-specific patterns, including the absence of overlap for rs56084453 near *ZNF35* (gamma and ductal cells) and rs62251653 near *SHQ1* (ductal cells) with their respective cell-type-specific chromatin accessibility peaks (Fig. 4C).

Notably, we identified conserved chromatin accessibility patterns across functionally distinct cell populations. *STIL* maintained accessible peaks in both insulin-producing beta cells and somatostatin-secreting delta cells, suggesting its potential role in co-regulating these endocrine lineages. Similarly, *ZSWIM5* exhibited chromatin accessibility across alpha, delta, and ductal cells, implying its potential involvement in bridging endocrine-exocrine communication.

The colocalization analysis further revealed significant correlations between eQTL effects of specific SNPs and GWAS effect sizes (Fig. 4D). Notably, we identified several SNPs exhibiting positive correlations between eQTL and GWAS effects in cell types where their associated genes were highly expressed in T2D, including rs12144920 (*STIL*) in beta and delta cells, rs1152020 (*ZSWIM5*) in alpha, delta, and ductal cells, and rs72950883 (*IL1RN*) in delta cells. Conversely, negative correlations were observed for SNPs where their genes showed T2D-specific low expression patterns: rs78676616 (*ANP32E*), rs3935296 (*IPP*), rs3806386 (*MLLT11*), and rs10170244 (*SLC23A3*) in delta cells; rs17331856 (*SNX4*) in gamma cells; and rs6742799 (*RBMS1*) in beta cells. These regulatory relationships demonstrate that disease-associated SNPs likely contribute to T2D pathogenesis through cell-type-specific mechanisms–either by enhancing gene expression in relevant cell types (positive eQTL effects) or, more importantly, by suppressing critical metabolic genes (negative eQTL effects) which may lead to dysfunction of essential metabolic pathways and ultimately disease progression.

To further validate the disease relevance of identified eGenes, we examined expression patterns of the nine key genes across four categories of pancreatic disease cell lines (Fig. 4E). These included: non-cancerous pancreas (ABMT9430), pancreatic neuroendocrine tumor (QGP1), adenosquamous carcinoma (KP3, L33), and 51 pancreatic adenocarcinoma lines (including PATU8988T and PANC1) (Table S8). Strikingly, *ZSWIM5* and *IL1RN* showed elevated expression across multiple disease contexts, whereas *ANP32E*, *IPP*, *MLLT11*, *SLC23A3*, *SNX4*, and *RBMS1* exhibited consistently reduced expression. This expression dichotomy directly aligns with the regulatory relationships observed in Fig. 4D: SNPs associated with highly expressed genes (*ZSWIM5*, *IL1RN*) demonstrated positive eQTL-GWAS effect correlations, while those linked to lowly expressed genes displayed negative correlations. This concordance across independent experimental models reinforces that the identified cis-regulatory mechanisms likely drive pathogenic gene expression programs in pancreatic diseases including T2D.

## Discussion

To our knowledge, this study represents the first comprehensive analysis of cis-eQTLs across all major pancreatic cell types at single-cell resolution, integrating scRNA-seq data with genotype information from a large cohort. We further mapped genetic effects on gene expression across all pancreatic cell types and constructed a pancreatic cell-type-specific cis-eQTL database called CTPeQTLs. By narrowing the scope from broadly identified GWAS risk loci to specific SNPs regulating gene expression within individual pancreatic cell populations, we substantially enhanced the precision of genetic analyses, enabling the identification of 371 high-confidence eGenes associated with T2D. Importantly, this approach revealed distinct cell-type-specific regulatory patterns and pinpointed candidate causal variants underlying known diabetes risk loci. Our findings thus fill a crucial gap in diabetes genetics by shifting the research paradigm from bulk tissue analysis toward detailed cell-type-specific investigation.

We developed a practical framework for identifying cell-type-specific cis-eQTLs, initially constructing a refined set of disease-relevant eQTLs from existing GWAS and eQTL datasets, subsequently mapping these eQTLs to specific pancreatic cell types via scRNA-seq. This analytical approach offers a valuable blueprint for future genetic studies aimed at dissecting the genetic architecture of complex diseases at single-cell resolution.

Critically, the identification of these cell-type-specific cis-eQTLs, culminating in the prioritization of nine high-confidence eGenes, provides new mechanistic insights into T2D pathogenesis by pinpointing where (specific cell types) and how (e.g. via chromatin accessibility) genetic risk variants functionally impact the pancreas. We identified nine genes across five pancreatic cell types as promising therapeutic targets for T2D: *STIL* in beta and delta cells; *ZSWIM5* in alpha, delta, and ductal cells; *IL1RN*, *ANP32E*, *IPP*, *MLLT11*, and *SLC23A3* in delta cells; *SNX4* in gamma cells; and *RBMS1* in beta cells, whose SNPs overlapped with chromatin accessibility peaks. The conserved overexpression of *ZSWIM5* and *IL1RN* across pancreatic disease models, coupled with their positive eQTL-GWAS correlations, strongly positions these factors as key regulators of pancreatic pathophysiology.

The associations of *RBMS1* (beta cells) and *STIL* (beta/delta) implicate potential genetic influences on beta cell proliferation/survival or RNA metabolism [54, 55]. The association of *SLC23A3* (delta cells) suggests a possible role in modulating oxidative stress responses [56]. *IL1RN* (delta cells) underscores the potential importance of localized islet inflammation via IL-1 signaling imbalance [57]. *SNX4* (gamma cells) links genetic variation to a process critical for insulin receptor trafficking efficiency [58]. Furthermore, the enrichment of key genes like *ANP32E*, *MLLT11*, and *IPP* specifically in delta cells hints at this population’s potential, yet previously underappreciated, role in integrating genetic risk signals that could affect overall islet homeostasis. These cell-type-specific hypotheses directly derived from our cis-eQTL mapping transform statistical associations into testable models, crucially informing potential therapeutic strategies.

Among these, previous studies provided experimental validation for SNX4 [58] and IL1RN [57] (Table S9). Specifically, SNX4 may influence diabetes development by regulating insulin receptor trafficking, while IL1RN hypomethylation contributes to disease progression through IL-1β/IL-1Ra axis imbalance in female patients. These findings support the robustness of our analytical pipeline and highlight the clinical relevance of these genes. In contrast, the majority of our prioritized candidates—*STIL*, *ZSWIM5*, *ANP32E*, *IPP*, *MLLT11*, *SLC23A3*, and *RBMS1*—currently lack direct experimental validation in T2D contexts. To guide future functional studies, we propose a prioritization strategy integrating multiple lines of evidence: (i) strong concordance between eQTL and GWAS effect sizes in disease-relevant pancreatic cell types; (ii) consistent dysregulation across independent pancreatic disease models; and (iii) enrichment in key T2D-related biological pathways. Based on these criteria, *RBMS1* (in beta cells) and *SLC23A3* (in delta cells) emerge as high-priority candidates due to their pronounced cell-type-specific effects on beta-cell identity and oxidative stress responses.

While experimental validation lies beyond the scope of the current study, these genes are well-suited for targeted functional testing in the future. Potential approaches include: (i) generating gene knockout or overexpression mouse models (global or beta-cell-specific) to assess their role in diabetes onset and progression; (ii) manipulating gene expression in established diabetic animal models to evaluate their therapeutic potential by monitoring phenotypic outcomes such as glucose tolerance, insulin secretion, and beta-cell mass; and (iii) performing gene knockdown or overexpression in relevant pancreatic cell lines (e.g. β-cell lines such as *INS-1* or *MIN6*, and α-cell lines such as *α-TC1*) to rapidly assess functional impacts on hormone secretion, stress responses, and key signaling pathways. Outlining such a multi-tiered roadmap not only bridges computational discovery and biological mechanism but also provides a foundation for translating these findings into precision therapies.

Building on this roadmap, it is also important to critically assess the data sources and methodological choices that underpin our current findings, as these factors shapes the robustness and interpretability of the results. Although blood-derived eQTL data may miss tissue-specific effects, its selection was driven by statistical power (*N* = 31684) unavailable in pancreatic datasets. Our framework mitigated this limitation by prioritizing eGenes expressed in pancreatic cells, validating variants against pancreatic ATAC-seq and revealing diabetes-relevant pathways. This suggests shared regulatory architecture enables blood eQTLs to inform pancreatic biology. Future pancreas-specific eQTL atlases will directly capture tissue-restricted variants, refining diabetes genetic models.

Expanding the genetic and ethnic diversity of study populations will also be critical in future research. Validation across larger, more diverse cohorts would confirm the robustness and global applicability of our identified cell-type-specific eQTLs. Furthermore, integrating multi-omics data, including proteomics and metabolomics, may provide deeper insights into how identified genetic variants impact cellular function and ultimately manifest as clinical phenotypes, supporting more precise therapeutic interventions.

## Conclusion

This study provides a comprehensive analysis of pancreatic cell-type-specific cis-eQTLs at single-cell resolution, identifying 328 eGenes linked to T2D pathogenesis. We integrated scRNA-seq, ATAC-seq, and GWAS data to reveal distinct regulatory patterns across endocrine and exocrine cells. This offers mechanistic insights into T2D-associated transcriptional dysregulation. Key findings highlight nine genes as promising therapeutic targets for T2D: *STIL* in beta and delta cells; *ZSWIM5* in alpha, delta, and ductal cells; *IL1RN*, *ANP32E*, *IPP*, *MLLT11*, and *SLC23A3* in delta cells; *SNX4* in gamma cells; and *RBMS1* in beta cells. To support further research, we built CTPeQTLs (https://ctpeqtls.netlify.app/), a public database integrating scRNA-seq data to explore pancreatic cell type-specific cis-eQTLs in diabetic versus non-diabetic cohorts, enabling comparative analysis of disease-associated regulatory variants. This work underscores the importance of cell-type-specific analyses in unraveling complex genetic contributions to diabetes and paves the way for targeted interventions.

**Key Points**

- Established a comprehensive catalog of pancreatic cell-type-specific cis-eQTLs linked to type 2 diabetes.
- Integrated single-cell RNA-seq, ATAC-seq, and GWAS data to reveal distinct cell-type-specific regulatory mechanisms.
- Identified nine key diabetes-associated genes (e.g. *STIL*, *IL1RN*, *ZSWIM5*) as potential targets in endocrine and ductal cells.
- We develop an interactive database (CTPeQTLs) enabling comparative analysis of cis-eQTLs in diabetic versus non-diabetic pancreata across cell types.
- Provided an innovative analytical framework for pinpointing cell-type-specific genetic regulators of complex diseases.

## Supplementary Material

Supplemental_Information_bbaf531

Supplemental_Information_bbaf531

## Data Availability

A publicly accessible website, CTPeQTLs (https://ctpeqtls.netlify.app/), has been developed to enable browsing of eGenes across various pancreatic cell types. The GWAS summary statistics of T2D are available on FinnGen consortium R10 at https://r10.finngen.fi/, under accession number finngen_R10_T2D. The full eQTL summary statistics are available on eQTLGen Consortium at https://molgenis26.gcc.rug.nl/downloads/eqtlgen/cis-eqtl/SMR_formatted/cis-eQTL-SMR_20191212.tar.gz. Single cell RNA-seq data are available at https://www.ebi.ac.uk/biostudies/arrayexpress/studies/E-MTAB-5061?query=E-MTAB-5061, https://www.ncbi.nlm.nih.gov/geo/query/acc.cgi?acc=GSE101207, https://www.ncbi.nlm.nih.gov/geo/query/acc.cgi?acc=GSE124742, https://www.ncbi.nlm.nih.gov/geo/query/acc.cgi?acc=GSE153855, https://www.ncbi.nlm.nih.gov/geo/query/acc.cgi?acc=GSE154126, https://www.ncbi.nlm.nih.gov/geo/query/acc.cgi?acc=GSE195986, https://www.ncbi.nlm.nih.gov/geo/query/acc.cgi?acc=GSE81608, https://www.ncbi.nlm.nih.gov/geo/query/acc.cgi?acc=GSE83139, https://www.ncbi.nlm.nih.gov/geo/query/acc.cgi?acc=GSE84133 and https://www.ncbi.nlm.nih.gov/geo/query/acc.cgi?acc=GSE86473. The merged single cell RNA-seq data are available at https://www.ebi.ac.uk/biostudies/studies/S-BSST2020?key=10f469dc-d7bd-4578-9540-241cdbbfcbef.Single cell ATAC-seq data are available at https://www.ncbi.nlm.nih.gov/geo/query/acc.cgi?acc=GSE194401. Database of SNP (dbSNP) variant call format file for the human reference genome GRCh38 (hg38) is available at http://ftp.broadinstitute.org/bundle/hg38/dbsnp_146.hg38.vcf.gz. All other data are available in the manuscript or the supplementary materials.

## Code availability

The code used to perform the analysis described in this study is available at https://github.com/xcmiao369/Cell-type-specific-cis-eQTLs-in-pancreatic-cell-types-identify-novel-type-2-diabetes-risk-genes.
