# Supplementary material for: Cell-type-specific cis-eQTLs in pancreatic cell types identify novel risk genes for type 2 diabetes
Source: Brief Bioinform. 2025 Oct 9;26(5):bbaf531. doi: 10.1093/bib/bbaf531 (PMC12510404; doi:10.1093/bib/bbaf531)
Supplement: Supplemental_Information_bbaf531 [file supplemental_information_bbaf531.docx]

**Cell-type-specific cis-eQTLs in pancreatic cell types identify novel risk genes for** **type 2 diabetes**


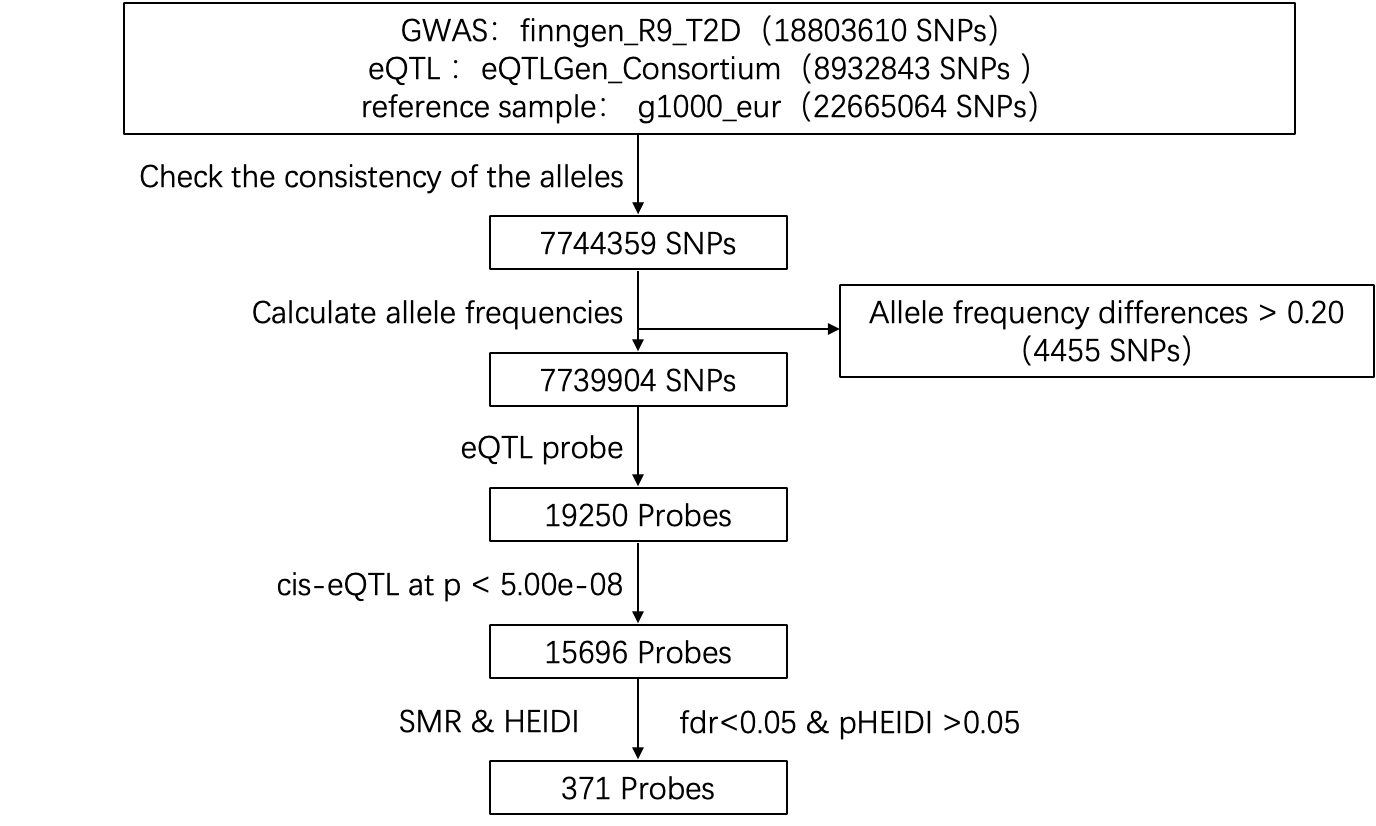


Figure S1. Summary-based Mendelian Randomization (SMR) analyses of eQTL dataset from eQTLGen Consortium.

Figure S2. Type 2 diabetic related eGene counts per chromosome.


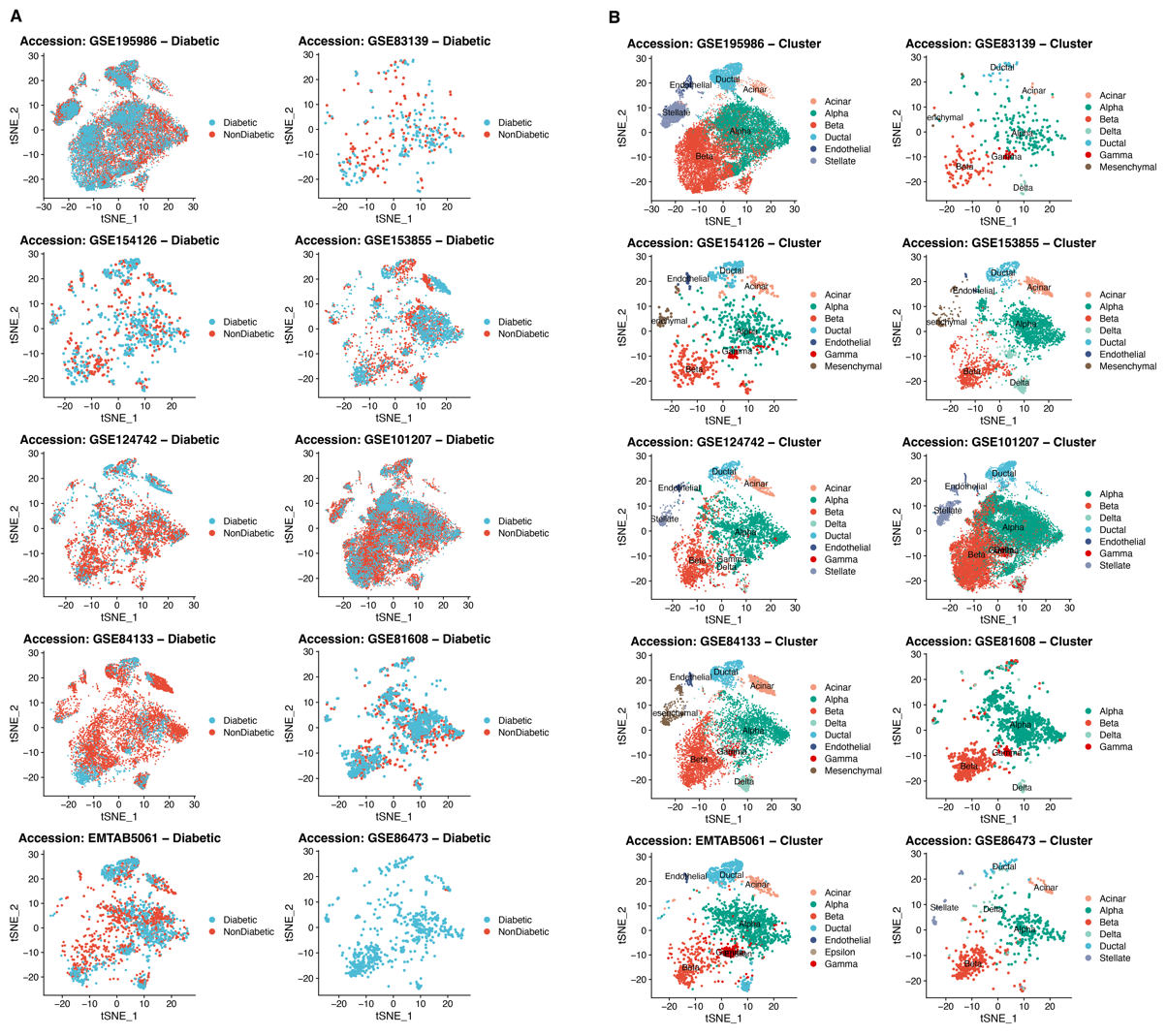


Figure S3. Cell-type analysis of human pancreatic cells of ten scRNA-seq datasets.


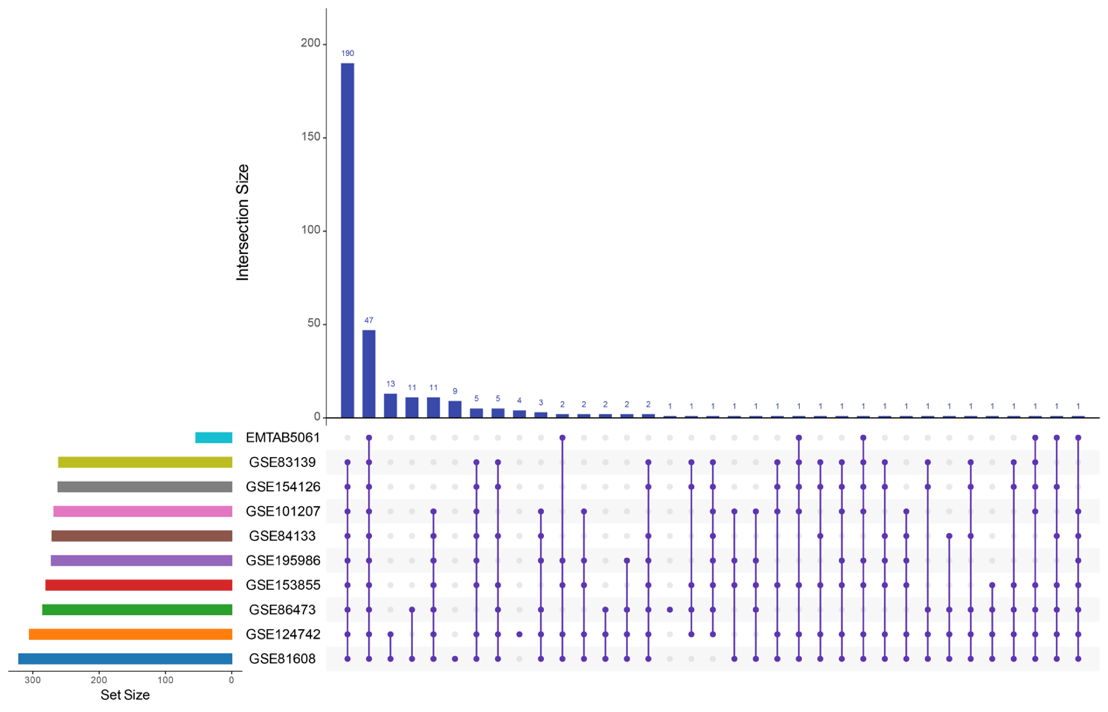


Figure S4. Multi-accession eGene overlap landscape in pancreatic cells.

Figure S5. Cell specific differentially expressed genes in diabetes.


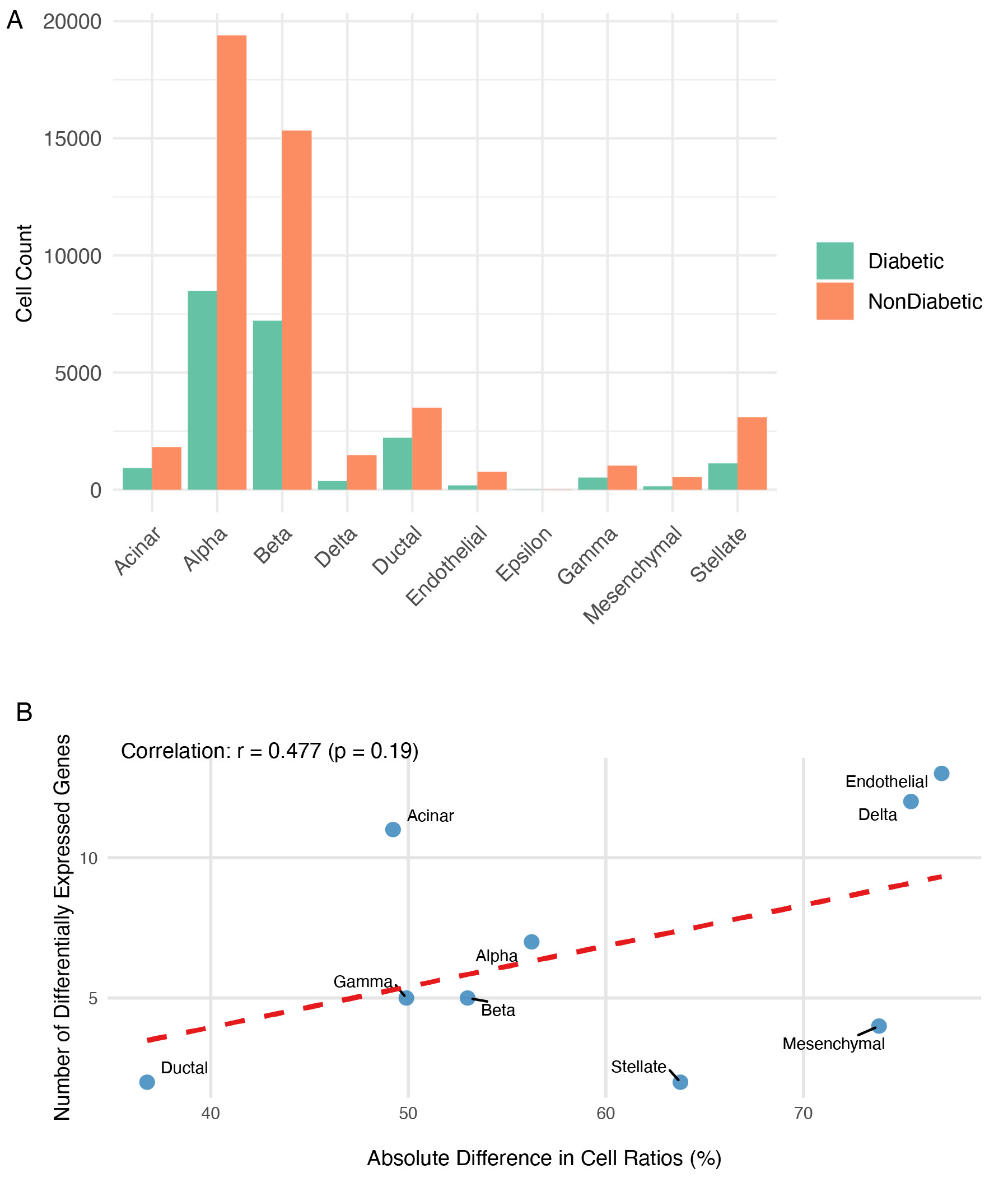


Figure S6. (A) Cell counts stratified by diabetic and non-diabetic status across pancreatic cell types. (B) Relationship between cell ratio differences and DEG counts across cell types.


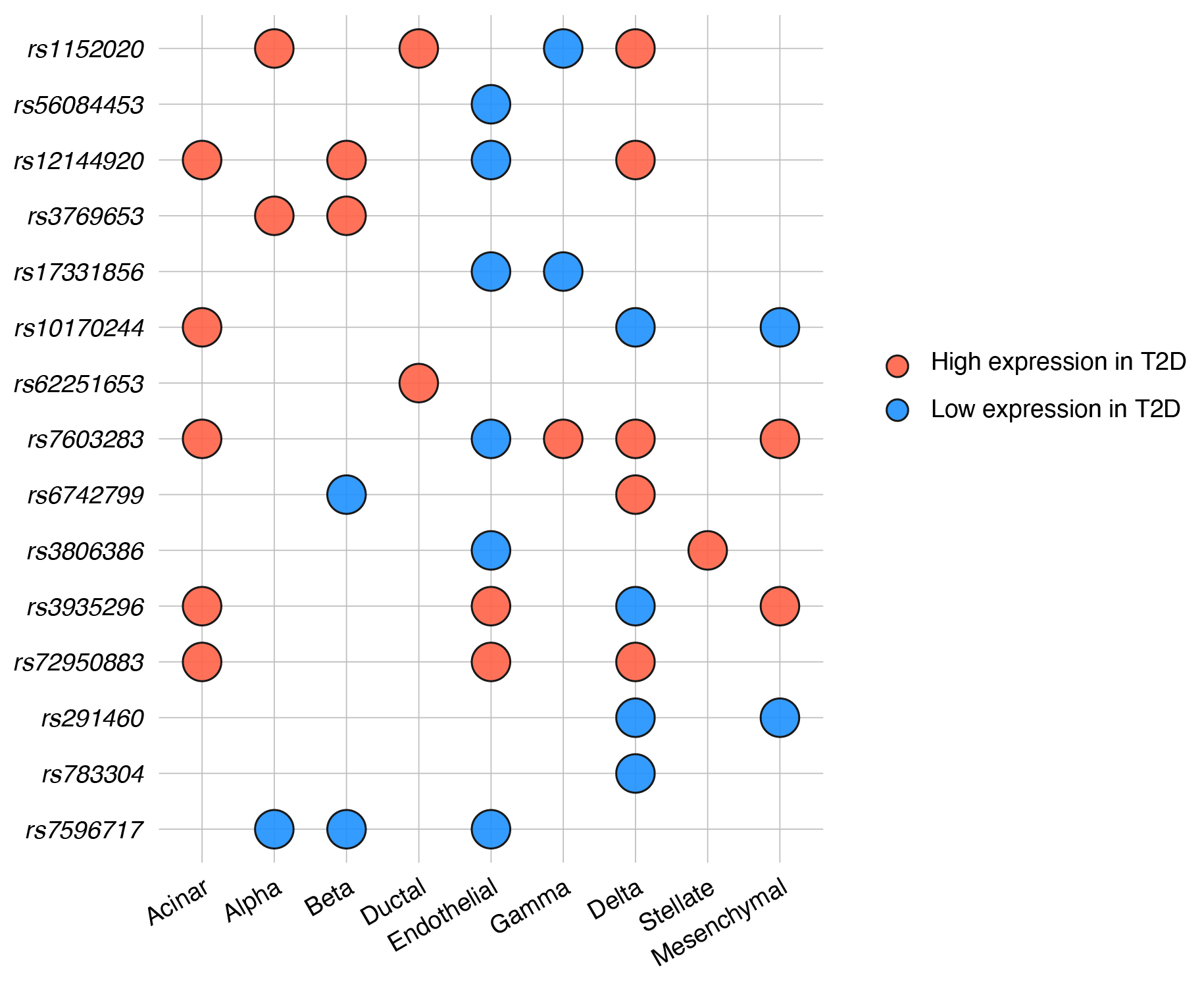


Figure S7. Summary of cell type-restricted SNP dysregulation in diabetic pancreas.

Figure S8. Type 2 diabetic related cell specific differentially expressed eGene counts per chromosome.
